# Ethanol as an electrolyte additive for alkaline zinc-air flow batteries

**DOI:** 10.1038/s41598-018-29630-0

**Published:** 2018-07-26

**Authors:** Soraya Hosseini, Siow Jing Han, Amornchai Arponwichanop, Tetsu Yonezawa, Soorathep Kheawhom

**Affiliations:** 1 Department of Chemical Engineering, Faculty of Engineering, Chulalongkorn University, Bangkok, 10330 Thailand; 2 Research Unit of Computational Process Engineering, Chulalongkorn University, Bangkok, 10330 Thailand; 3 Department of Chemical Engineering, Faculty of Engineering, University Technology PETRONAS, Seri Iskander, Perak 32610 Malaysia; 4 Division of Materials Science and Engineering, Faculty of Engineering, Hokkaido University, Kita 13 Nishi 8, Sapporo, Hokkaido 060-8628 Japan

## Abstract

Zinc-air flow batteries exhibit high energy density and offer several appealing advantages. However, their low efficiency of zinc utilization resulted from passivation and corrosion of the zinc anodes has limited their broad application. In this work, ethanol, which is considered as an environmentally friendly solvent, is examined as an electrolyte additive to potassium hydroxide (KOH) aqueous electrolyte to improve electrochemical performance of the batteries. Besides, the effects of adding different percentages of ethanol (0–50% v/v) to 8 M KOH aqueous electrolyte were investigated and discussed. Cyclic voltammograms revealed that the presence of 5–10% v/v ethanol is attributed to the enhancement of zinc dissolution and the hindrance of zinc anode passivation. Also, potentiodynamic polarization and electrochemical impedance spectroscopy confirmed that adding 5–10% v/v ethanol could effectively suppress the formation of passivating layers on the active surface of the zinc anodes. Though the addition of ethanol increased solution resistance and hence slightly decreased the discharge potential of the batteries, a significant enhancement of discharge capacity and energy density could be sought. Also, galvanostatic discharge results indicated that the battery using 10% v/v ethanol electrolyte exhibited the highest electrochemical performance with 30% increase in discharge capacity and 16% increase in specific energy over that of KOH electrolyte without ethanol.

## Introduction

Zinc-air batteries are attractive for various future energy applications due to their low cost, high safety, high specific energy density, and environment-friendliness^1–3^. In recent years, zinc-air flow batteries, also known as zinc-air fuel cells, have been demonstrated. These batteries can be swiftly refueled using zinc powder and granules^4^. Nevertheless, low efficiency of zinc utilization limits their extensive use^5^.

An electrolyte plays a significant role in battery electrochemistry that affects the transport properties of the active species, energy and power density of batteries. Enormous research effort has been carried out to enhance the battery performance by improving the electrolyte as this approach is simple and does not affect the specific energy of the battery^6–8^.

Alkaline aqueous electrolytes such as potassium hydroxide (KOH)^9^, sodium hydroxide (NaOH)^10^ and lithium hydroxide (LiOH)^11^ are widely implemented in various types of batteries^3^. KOH is extensively applied due to the desirable ionic conductivity of K^+^ (73.5 Ω^−1^ cm^2^/equiv) compared to Na^+^ (50.11 Ω^−1^ cm^2^/equiv) and Li^+^ (38.7 Ω^−1^ cm^2^/equiv). Previously, the influence of KOH concentration has been studied and reported^12,13^. Zinc oxide solubility increases with the increasing of KOH concentration. However, the increase in KOH concentration decreases the electrode potential. Also, the rise of KOH concentration leads to its high viscosity, consequently, a decrease in the transfer rate of hydroxide ion. The optimum level of KOH was found to be 6–8 M for both ionic conductivity and exchange current associated with reaction kinetics.

There are some practical limitations to be aware of in using KOH aqueous electrolyte^8^. In particular, the self-corrosion of the zinc anode leads to a decrease of discharge capacity. Also, the precipitation of discharged products on the active zinc anode leads to undesirable passivation effect.

Neutral electrolytes exhibited a safer and more robust alternative to traditional alkaline aqueous electrolyte^14–17^. However, their ionic conductivities are by far lower than conventional alkaline electrolytes. Besides, non-aqueous electrolytes such as ionic liquids have been studied^18,19^. Nevertheless, their high cost and sluggish reaction kinetic of oxygen reduction reaction cathode hinder their practical use^20^.

There are some reports about introduction of additives into alkaline aqueous electrolytes to enhance battery performances. The use of electrolyte additives is a simple, economical, and effective approach. Silicate (SiO_3_^2−^) have been studied in alkaline electrolytes^21^. Besides, it was reported that sodium dodecyl benzene sulfonate in alkaline electrolyte can minimize the passivation of the zinc active surface and improve the discharge capacity^22^. Also, the use of ionic liquids as electrolyte additives has been reported^23^.

Ethanol is an alcohol and considered a green solvent. In addition, it is a polar protic solvent containing a labile H^+^. It has two carbon atoms in its chain, and the chain ends in OH group. Also, ethanol can completely dissolve in water. Lee *et al*.^24^ reported that alkoxide ions, resulted from the transformation of alcohols in alkaline solutions, can compete with hydroxide ions for coordination to Zn^2+^ ions. Thus, the formation of zinc oxide (ZnO) could be significantly suppressed by adding alcohols. Though this approach is simple but practical, there are only a few studies on the application of ethanol as an electrolyte additive.

This work aims at enhancing electrochemical performances of zinc-air flow batteries by introducing ethanol in 8 M KOH aqueous electrolyte to suppress corrosion and passivation effects of granular zinc anodes. Various percentages of ethanol (0–50% v/v) were investigated to identify the optimum ratio of KOH/Ethanol. Cyclic voltammetry, electrochemical impedance spectroscopy, potentiodynamic polarization measurements are used to investigate the electrochemical characteristics and mechanisms of zinc oxidation/reduction. Also, the performances of the zinc-air flow batteries using these electrolytes were examined and discussed.

## Experimental

### Chemical and Materials

Nickel (Ni) foam with a purity of 99.97%, 100 pores per inch (PPI) and 1 mm thick, used as the cathode current collector, was purchased from Qijing Trading Co., Ltd. 100 mesh of woven wire 304 stainless steel, used as the anode current collector, was purchased from Alikafeii Trading Co., Ltd. Zinc granules with a purity of 99.99% and an average diameter of 0.8 mm, purchased from Sirikul Engineering Ltd., Part., were used as the anode. KOH pellets (99%) and ethanol (99.8%), purchased from CT Chemical Co., Ltd., were used to prepare the electrolytes. Manganese(IV) oxide (MnO_2_, 5 *μ*m 99.99%, Sigma-Aldrich), carbon black (Vulcan® XC-72, Cabot Corporation), and poly(tetrafluoroethylene) (PTFE powder, 1 *μ*m, Sigma-Aldrich) were used to prepare the cathode. Poly(vinyl butyral) (PVB), purchased from Sigma-Aldrich, was used as a binder. Whatman filter paper No. 1 (Sigma-Aldrich) and poly(vinyl acetate) (PVAc) (TOA Paint Public Co., Ltd.) were used to prepare the separator. Graphite foil, purchased from Mineral Seal Corporation, was employed in electrochemical property characterization. All chemicals were used as received and without any further purification.

### Electrode and battery fabrication

**Figure 1:**
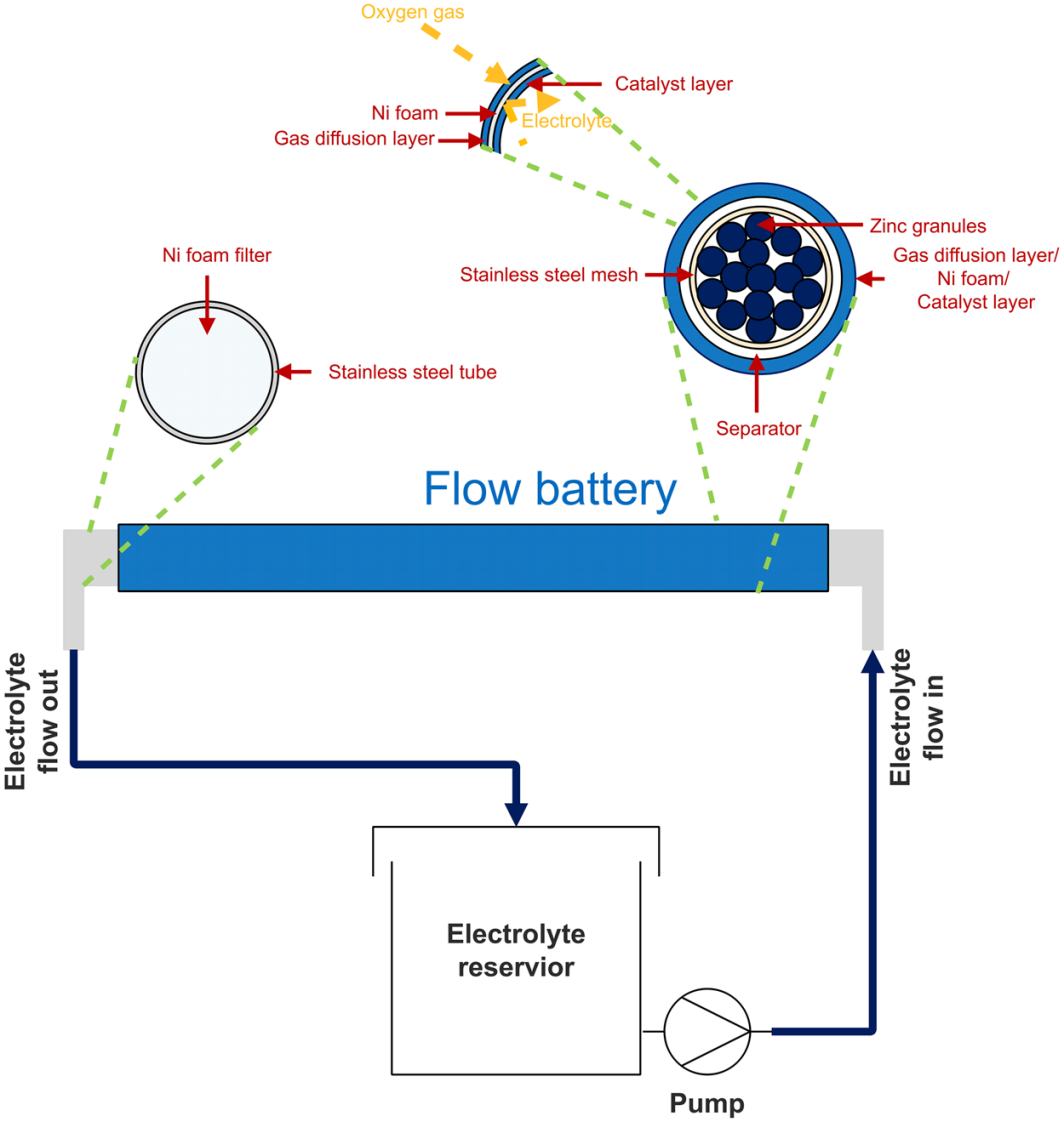
A schematic diagram of the zinc-air flow batteries.

The behavior and performance of zinc-air flow batteries were examined using home-made batteries. The schematic diagram of the batteries is shown in Fig. 1. A stainless steel mesh cylinder (0.5 cm outer diameter), connected to stainless steel tube at both ends, was used as the anode current collector. The cylinder was covered with the separator prepared by casting 2 g of 24 wt.% PVAc solution in water over both sides of a filter paper (1.6 cm × 7 cm). After casting each side, the sample was then dried in an oven at 55 °C for 10 min. Afterward, the cylinder was covered with a cathode sheet, composed of three layers namely a gas diffusion layer, a cathode current collector, and a catalyst layer. The catalyst layer is placed in contact with the separator. The active area of the cathode was 10 cm^2^. Ni foam was used as the cathode current collector. To fabricate the catalyst layer, a slurry prepared by mixing 2 g of MnO_2_, 7 g of carbon black, 1 g of PTFE powder, and 0.45 g of PVB in 10 ml of ethanol, was deposited on one side of the Ni foam. Totally 0.5 g of the slurry was deposited on the Ni foam. To prepare the gas diffusion layer, the other side of the Ni foam was coated with a slurry prepared by mixing 3 g of carbon black, 7 g of PTFE powder, and 0.5 g of PVB in 10 ml of ethanol. Totally 0.5 g of the slurry was deposited on this side of the Ni foam. The coated Ni foam was then heat-pressed at 350 °C for 5 min using a hot press machine. The gas diffusion layer exhibits hydrophobicity and keeps the electrolyte inside the cell while allowing oxygen gas to diffuse to the catalyst layer. Besides, the hydrophobicity of the gas diffusion layer prevents leakage of the electrolyte and water flooding in the cathode.

KOH aqueous solutions (8 M KOH) containing 0–50% v/v ethanol were used as the electrolyte. During discharge, the electrolyte was circulated through the cell at a circulation rate of 20 mL/min using a peristaltic pump.

### Characterization and measurement

The electrochemical characteristics of the electrolytes were examined using electrochemical impedance spectroscopy (EIS), cyclic voltammetry (CV) and potentiodynamic polarization measurements using a potentiostat/galvanostat with impedance measurement unit (AMETEK, PAR VersaSTAT 3A). These experiments were carried out using a three-electrode configuration cell with a platinum plate (2 cm × 2 cm) as the counter electrode, and a mercury/mercury oxide (Hg/HgO) electrode as the reference electrode. Initially, CV experiments were performed using a stainless steel mesh tube (2 mm inner diameter) or graphite foil (1 cm × 1 cm) as the working electrode. Besides, the effects of ethanol in the electrolyte were studied by CV using 0.5 g zinc electroplated on graphite foil (1 cm × 1 cm) as the working electrode. Finally, the effects of ethanol/KOH in the system using a stainless steel current collector, which is similar to the flow batteries studied in this work, were investigated by CV, EIS, and potentiodynamic polarization measurements using zinc granules with total surface area 20 mm^2^ in a stainless steel tube (2 mm inner diameter) as the working electrode. In most cases, CV experiments were carried out from −1.8 to 0.6 V with a scan rate of 0.05 V/s unless otherwise specified. In the forward scan referred as the anodic trace, the potential was swept from the initial potential of −1.8 V to the switching potential of 0.8 V. The scan direction was then reversed, and the potential was swept back to −1.8 V referred as the cathodic trace or reverse scan. The EIS measurements were carried out at the potential 0 V (vs. OCV) with the frequency range from 0.01 Hz to 100 kHz with alternate current (AC) amplitude of 10 mV. The potentiodynamic polarization measurements were carried out using a scan rate of 0.065 V/s.

The flow batteries were fabricated and operated using the electrolyte circulation of 20 mL/s. The performance of the batteries was examined using a battery analyzer (Battery Metric, MC2020).

## Results and Discussion

The oxidation of zinc, also known as zinc dissolution, is the primary reaction determining the electrochemical performance of the zinc anode. Zinc is amphoteric, and its oxidation product can dissolve in an alkaline solution to form soluble zincate ion $({\rm{Zn}}{({\rm{OH}})}_{4}^{2-})$^25^. Though, the flow batteries studied in this work employed a stainless steel mesh tube as the anode current collector, the cyclic voltammograms of the stainless steel mesh tube and the graphite foil in 8 M KOH electrolyte was examined. Then, the effects of ethanol in the electrolyte were studied using the graphite foil.

**Figure 2:**
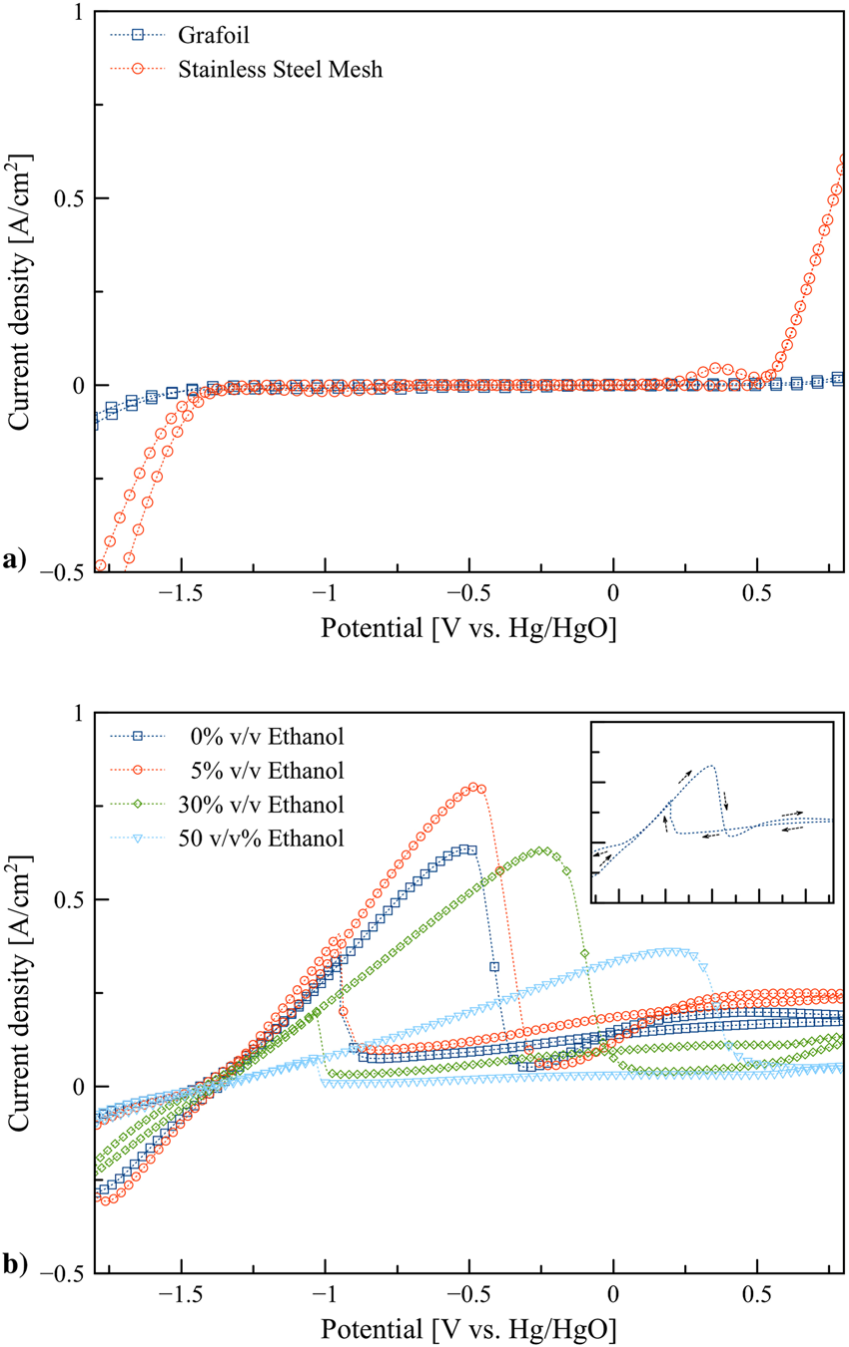
Cyclic voltammograms: (**a**) graphite foil and stainless steel mesh tube in 8 M KOH electrolytes at scan rate 0.05 V/s, and (**b**) 0.5 g of Zn electroplated on graphite foil in 0, 5, 30, and 50% v/v ethanol/KOH electrolytes at scan rate 0.05 V/s.

Figure 2a displays cyclic voltammograms of the graphite foil and stainless steel mesh tube in 8 M KOH. For both forward and reverse scans, no peaks were observed for the graphite foil at the potentials between −1.4 to 0.5 V vs. Hg/HgO. The onset potential for oxygen evolution was at 0.5 V vs. Hg/HgO. Besides, hydrogen evolution occurred below −1.4 V vs. Hg/HgO. In the same manner, the onset potentials for oxygen and hydrogen evolution for the stainless steel mesh tube were similar to those of the graphite foil. Nevertheless, the evolution of oxygen and hydrogen took place slightly on the graphite foil than those on the stainless steel mesh tube. Besides, one anodic peak at 0.25–0.5 V vs. Hg/HgO was observed for the stainless steel mesh. This peak is related to the oxidation of chromium (Cr) on the surface of stainless steel^26^. The results indicate that in the range −1.4 to 0.5 V vs. Hg/HgO, the graphite foil is inert. Thus, the graphite foil was employed as the substrate to examine the effects of ethanol in 8 M KOH solution. The comparison between cyclic voltammograms of various ratios of ethanol/KOH measured with a scan rate of 0.05 V/s was carried out using 0.5 g zinc electroplated on the graphite foil as the working electrode. Figure 2b presents the cyclic voltammograms in 0, 5, 30, and 50% v/v ethanol/KOH electrolytes. In all cases, the onset potential of zinc dissolution occurred around −1.4 V vs. Hg/HgO on the forward scan. This peak corresponds to zinc dissolution or zincate formation as shown in reaction (1). Also, the formation of ${\rm{Zn}}{({\rm{OH}})}_{3}^{-}$ ion resulted from the OH^−^ ion depletion at the electrode surface can also occur through reaction (2)^27,28^. The zincate formation plays the most crucial role in zinc dissolution and hence determining performance of the batteries.

1 $${\rm{Zn}}+4{{\rm{OH}}}^{-}\rightleftharpoons {\rm{Zn}}{({\rm{OH}})}_{4}^{2-}+2{{\rm{e}}}^{-}$$

2 $${\rm{Zn}}+3{{\rm{OH}}}^{-}\rightleftharpoons {\rm{Zn}}{({\rm{OH}})}_{3}^{-}+2{{\rm{e}}}^{-}$$

The presence of 5% v/v ethanol increased the peak current density leading to higher zinc dissolution. Nevertheless, adding a higher amount of ethanol, the peak significantly decreased and shifted toward more positive potential indicating the decrease of the zinc dissolution. Besides, on the reverse scan, the oxidation peaks were observed at the same range of potentials appeared on the forward scan. The oxidation peaks on the forward scan may be resulted from a diffusive limiting of hydroxide ions. Thus, on the reverse scan, zinc continues to oxidize in the oxidation potential range. The current density reached zero and switched to reduction at −1.4 V vs. Hg/HgO, which is also the onset potential of zinc dissolution.

**Figure 3:**
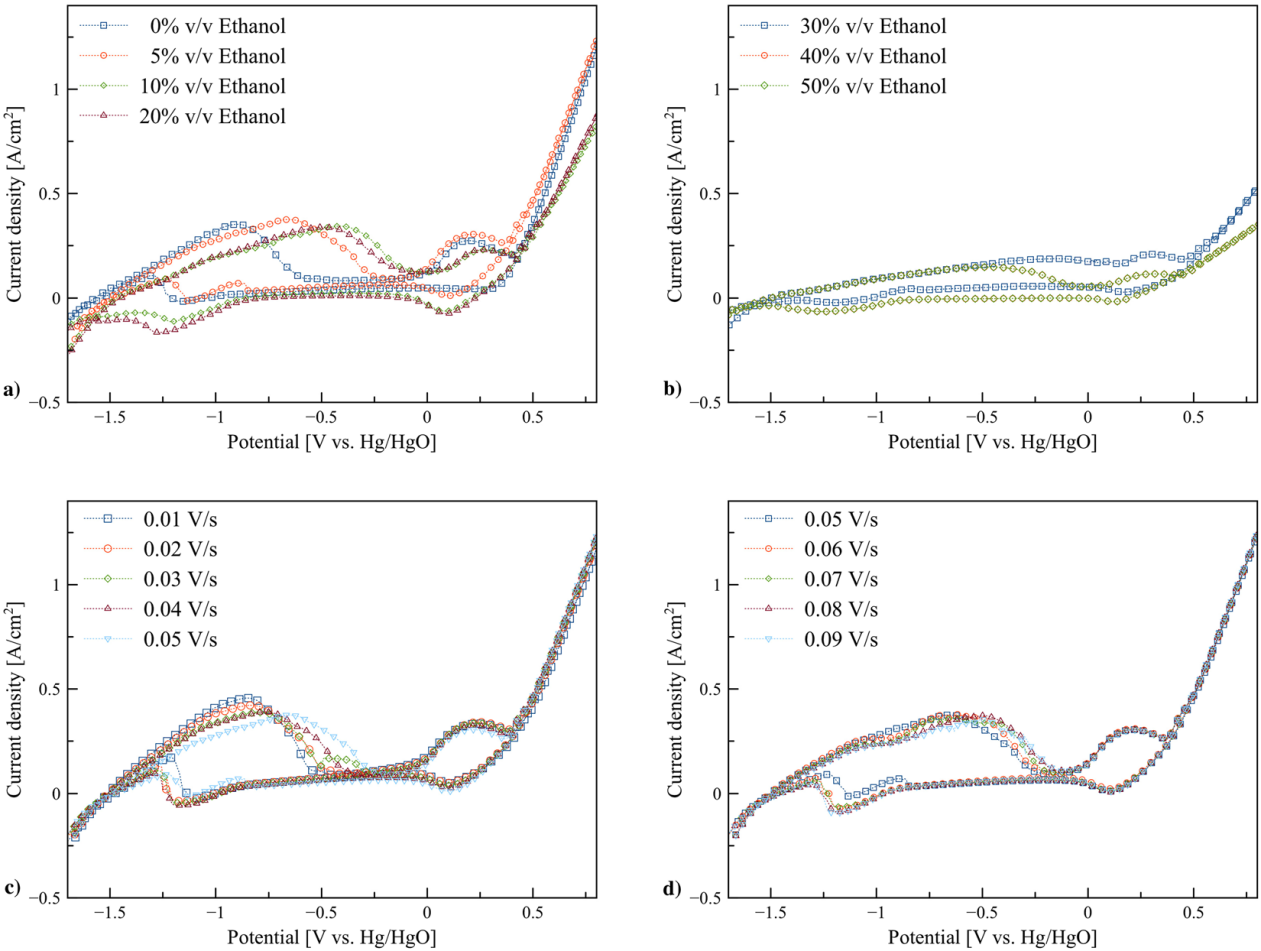
Cyclic voltammograms: (**a**) Zn in 0, 5, 10, and 20% v/v ethanol/ KOH electrolytes at scan rate 0.05 V/s, (**b**) Zn in 30, 40, and 50% v/v ethanol/KOH electrolytes at scan rate 0.05 V/s, (**c**) Zn in 5% v/v ethanol/KOH electrolyte at various scan rates (0.01, 0.02, 0.03, 0.04, and 0.05 V/s), and (**d**) Zn in 5% v/v ethanol/KOH electrolyte at various scan rates (0.05, 0.06, 0.07, 0.08, and 0.09 V/s).

The dissolution of zinc in ethanol/KOH electrolytes was further examined using the stainless steel mesh tube as this material was used as the anode current collector in the flow batteries. Figure 3a and b show the cyclic voltammograms of the electrolytes containing ethanol ranging from 0% to 50% v/v in 8 M KOH solution. The results present several cathodic and anodic peaks. Two upward anodic (oxidation) peaks were observed. The first peak around −0.8 to −0.4 V vs. Hg/HgO with onset potential around −1.4 V vs. Hg/HgO is assigned to zinc dissolution. The ethanol content significantly affected the shape of cyclic voltammograms. The peak of zinc dissolution increased with the presence of ethanol in range of 5% to 20% v/v. Nevertheless, above 20% v/v ethanol, the peak significantly dropped and vanished. Also, by adding ethanol, the anodic peaks were shifted toward more positive potential. The possible reason for the shifting may be due to the limit of the diffusion of discharge products or formation alkali metal alkoxides. Alkali metal alkoxides are produced by reactions between alcohols and alkaline solution as presented in reaction (3)^29^. Different ethanol concentrations affected the rate of zinc dissolution due to the formation of by-products such as potassium ethoxide.

3 $${\rm{MOH}}+{\rm{ROH}}\rightleftharpoons {\rm{ROM}}+{{\rm{H}}}_{2}{\rm{O}}$$

The slopes at the right end from 0.5 V onward corresponds to the decomposition of hydroxide ion (OH^−^) and formation of oxygen (O_2_) and water (H_2_O) according to the oxidation reaction^30^ as shown in reaction (4). In general, a shift of this slope due to the variation of concentration and activity of H^+^ and OH^−^ is expected. By the addition of ethanol, a decrease of this slope was observed. Besides, the highest slope was observed for 0% v/v ethanol due to the highest concentration of OH^−^. On the left end of the reverse scan, a sharp decrease in the current was detected due to the hydrogen evolution reaction in alkaline media^31^.

4 $$4{{\rm{OH}}}^{-}\rightleftharpoons {{\rm{O}}}_{2}+2{{\rm{H}}}_{2}{\rm{O}}+4{{\rm{e}}}^{-}$$

The effects of scan rate from 0.01 to 0.09 V/s for the electrolyte containing 5% v/v ethanol are shown in Fig. 3c and d. The zinc dissolution peaks are shifted toward positive potential with an increase in the scan rate. The shifting of the peak potential confirmed the irreversibility of the electron transfer reaction. For the irreversible systems, the peak current and the position of the potential are influenced by reaction kinetics and mechanisms. Various mechanisms were suggested for zincate formation in the literature^32,33^. Zinc dissolution process can be devided into a number of elementary first order reactions. Different reaction mechanisms were proposed with three^34,35^ or four elementary reactions^32,36^.

5 $${\rm{Zn}}+{{\rm{OH}}}^{-}\rightleftharpoons {\rm{Zn}}({\rm{OH}})+{{\rm{e}}}^{-}$$

6 $${\rm{Zn}}({\rm{OH}})+{{\rm{OH}}}^{-}\rightleftharpoons {\rm{Zn}}{({\rm{OH}})}_{2}^{-}$$

7 $${\rm{Zn}}{({\rm{OH}})}_{2}^{-}+{{\rm{OH}}}^{-}\rightleftharpoons {\rm{Zn}}{({\rm{OH}})}_{3}^{-}+{{\rm{e}}}^{-}$$

8 $${\rm{Zn}}{({\rm{OH}})}_{3}^{-}+{{\rm{OH}}}^{-}\rightleftharpoons {\rm{Zn}}{({\rm{OH}})}_{4}^{2-}$$

It was reported that the oxidation of ${\mathrm{Zn}(\mathrm{OH})}_{2}^{-}$ is found to be rate-limiting step. At low scan rate (slow electron transfer), zinc anode has enough contact time with hydroxide ions to produce ${\mathrm{Zn}(\mathrm{OH})}_{4}^{2-}$. In contrary, by increasing the scan rate, the electron transfer steps may be eliminated leading to precipitation of ZnO on the zinc surface.

**Figure 4:**
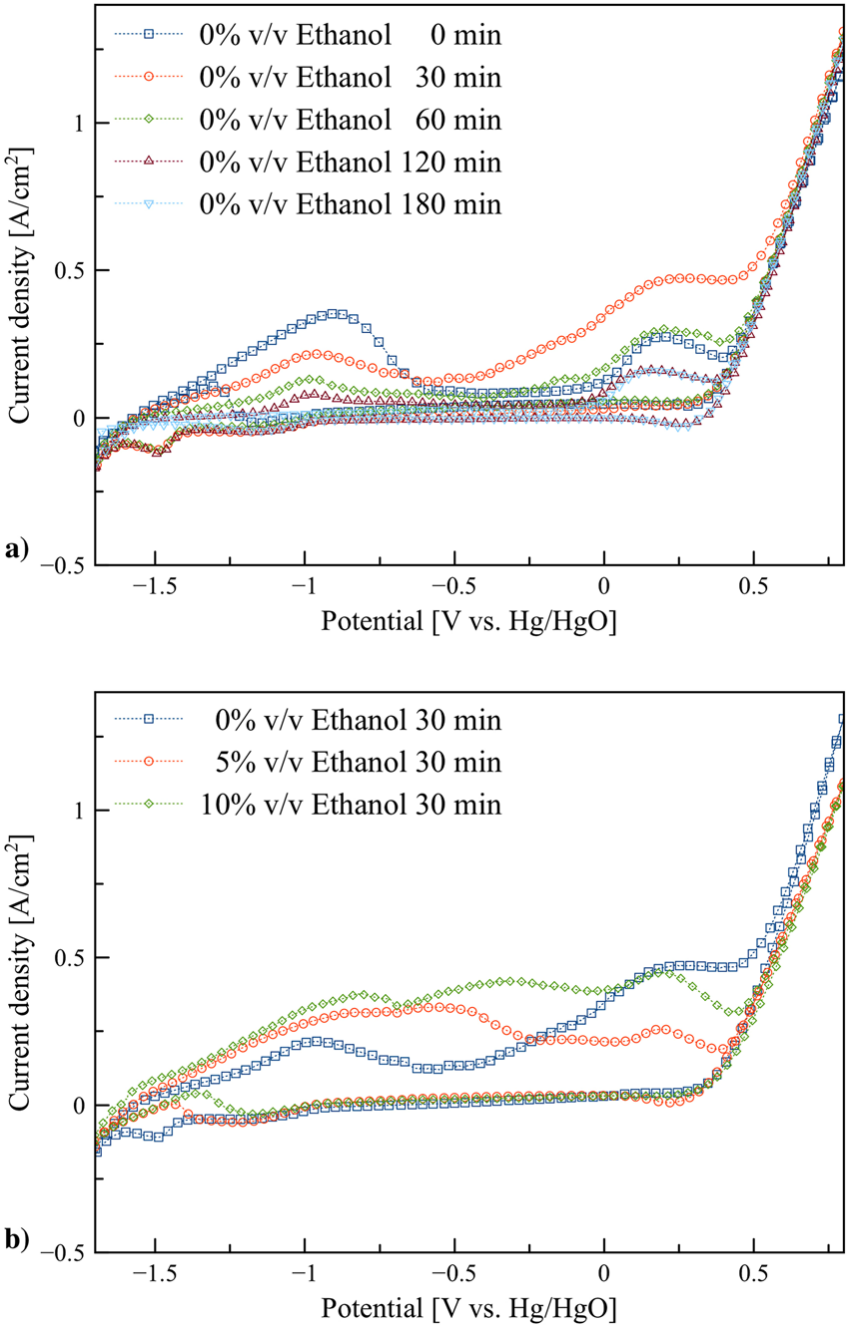
Cyclic voltammograms: (**a**) Zn in 0% v/v ethanol/KOH electrolytes at scan rate 0.05 V/s after immersed for 0–180 min, and (**b**) Zn in 0–10% v/v ethanol/KOH electrolytes at scan rate 0.05 V/s after immersed for 30 min.

An examination of cyclic voltammetry for 0% v/v ethanol after immersed in the electrolyte for 0–180 min was studied. As shown in Fig. 4a, the size of anodic peaks substantially changed with the immersion time. The significant decrease was observed after 30 min. Besides, when the immersion time was prolonged above 180 min, the size of the characteristic peaks remained the same. At 30 min, the maximum formation of ZnO was reached and the peaks size decreased and stabilized after 120 min due to the formation of passivation layers hindering further zinc dissolution.

Figure 4b shows a comparison of cyclic voltamograms for 0%, 5% and 10% ethanol electrolytes at 30 min immersion time. In comparison to 0% v/v ethanol electrolyte, the electrolyte containing 5% v/v and 10% v/v ethanol exhibited improved zinc dissolution. The dissolution of zinc increased for both volume ratios of ethanol whilst 0% v/v ethanol exhibited the lowest zinc dissolution.

**Figure 5:**
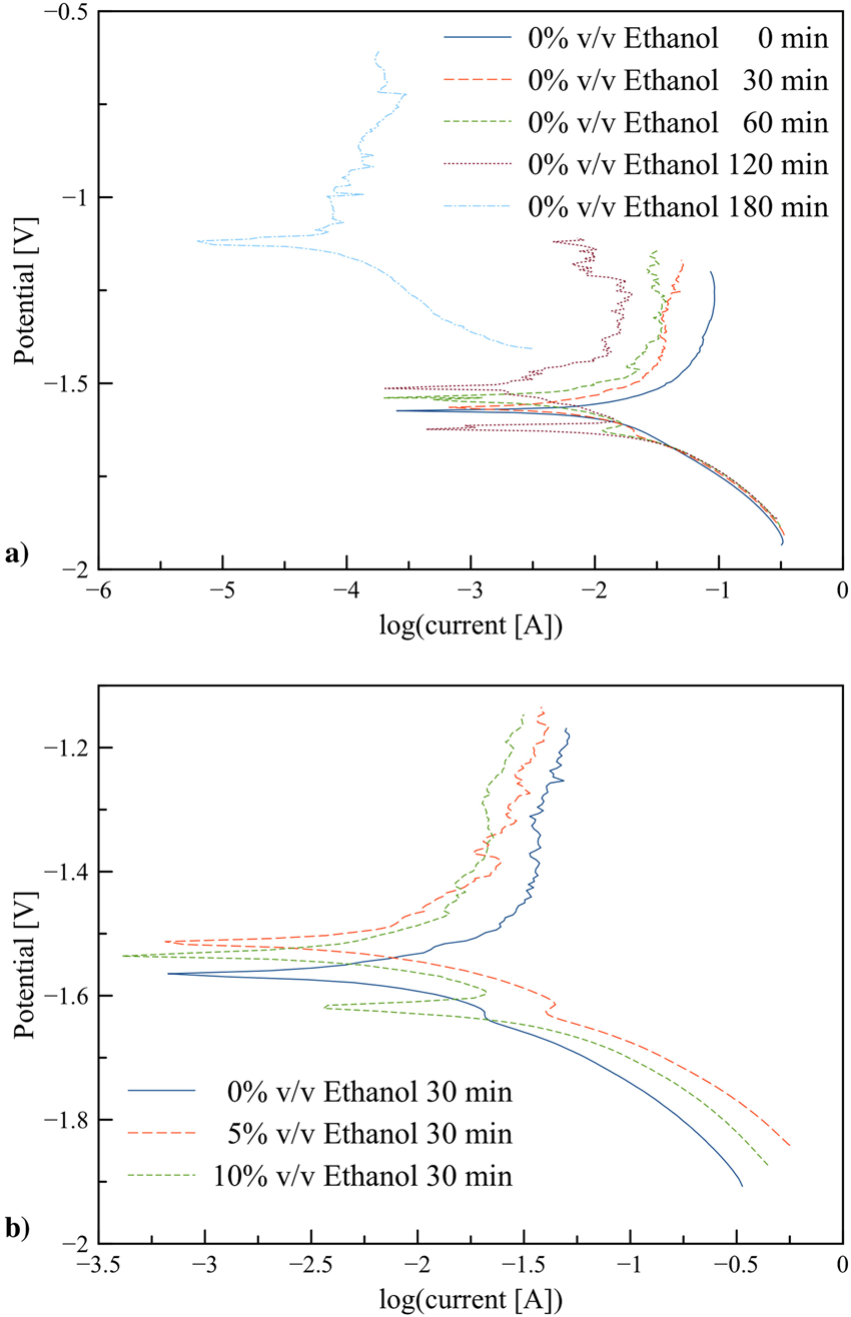
Potentiodynamic polarization characteristics of the electrolytes using a scan rate 0.065 mV/s: (**a**) 0% v/v ethanol with immersion time 0–180 min, and (**b**) 0, 5, and 10% v/v ethanol electrolytes with immersion time 30 min.

The evaluation of time dependent changes of potentiodynamic polarization characteristics of 0% v/v ethanol was carried out for the immersion time 0 to 180 min as presented in Fig. 5a. At the beginning of immersion (0 min), the sample exhibited the lowest rest potential. At longer immersion time, the rest potential shifted upward. The results indicated that at the beginning the anode was fresh and showed the highest anodic activity. The anodic potential gradually shifted to more positive values by increasing time, indicating the formation of ZnO layer acting as a passivation layer and supprssing zinc dissolution. In comparison, the cathodic hydrogen evolution reaction was not significantly affected by increasing immersion time from 0 to 180 min.

Figure 5b shows potentiodynamic polarization characteristics of 0%, 5%, 10% v/v ethanol electrolytes with immersion time 30 min. The similar trends were observed in all cases. The hydrogen evolution observed in cathodic branches was enhanced for the electrolytes containing ethanol. It was reported the effects of hydrogen absorbed on metal surfaces on anodic polarization in several aspects including retarding the formation of passivating film, changing the condition of electrode surface, decreasing the resistance toward charge transfer and ion diffusion, and increasing the capacitance^37,38^.

The anodic reaction can be examined using the Tafel equation as shown in (9)^39^. The exchange current density (*i*_0_) is determined by plotting the voltage versus the logarithm of current. The Tafel slope and intercept are related to the electron transfer coefficient and exchange current density, respectively. The parameters *α* and *i*_0_ can be estimated by regression analysis. The corrosion potential *E*_corr_ and the corrosion current *I*_corr_ can be obtained by Tafel extrapolation of the anodic and cathodic branches.

9 $$\eta =\frac{-RT}{\alpha nF}\,\mathrm{ln}({i}_{0})+\frac{RT}{\alpha nF}\,\mathrm{ln}(i)$$

The highest electron transfer coefficient with lower potential corrosion was observed for 5% v/v ethanol. This observation is consistent with CV results. It can be concluded that ethanol was significantly responsible for the high dissolution rate of zinc anode and zincate formation.

The asymmetry of cathodic and anodic branches of the corresponding Tafel plots indicated an irreversible electrochemical reaction where the cathodic branch is slightly larger than the anodic branch. The asymmetry may be associated with major differences in the short-range solvent polarization between the oxidized and reduced species. The estimated parameters by Tafel plots are listed in Table 1. Both *E*_corr_ and *I*_corr_ of the electrolytes containing ethanol are more postive than those of 0% v/v ethanol electrolyte. Therefore, the electrolytes containing ethanol provided lower corrosion rate.

**Table 1:** Parameters of Tafel analysis for the zinc anodes in 0%, 5% and 10% v/v ethanol electrolytes.

Electrolyte	*E*_corr_ (V)	*I*_corr_ (A)	*α*	log(*i*_0_) (A)	slope (mV/dec)
0% v/v	−1.565	7E-4	0.227	−9.65	13.27
Ethanol					
5% v/v	−1.513	6E-4	0.339	−14.66	8.87
Ethanol					
10% v/v	−1.536	4E-4	0.297	−12.72	10.11
Ethanol					

**Figure 6:**
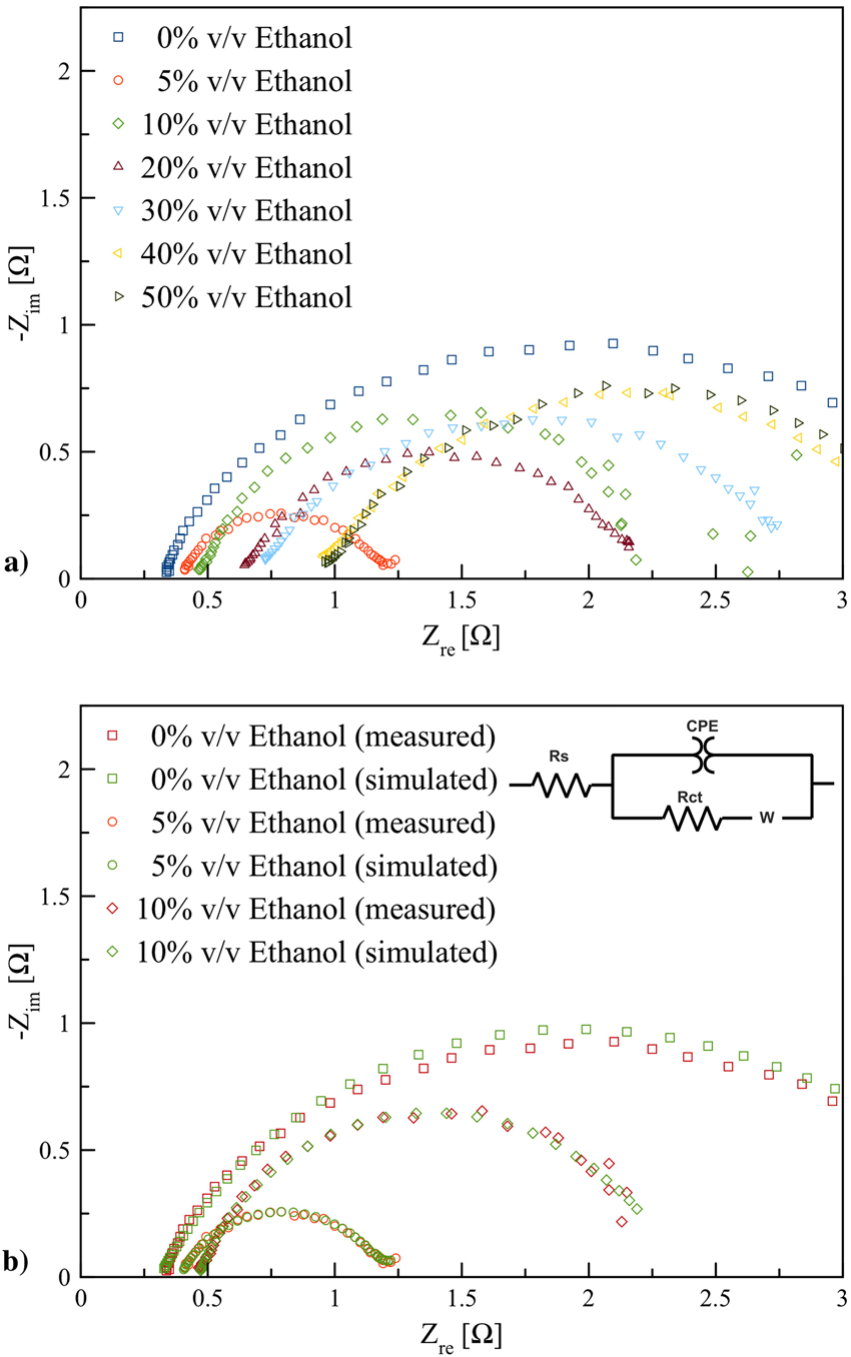
(**a**) Nyquist plot of EIS performed at the potential 0 V in the frequency range 0.01 Hz to 100 kHz with AC amplitude of 10 mV of the electrolytes containing 0–50% v/v ethanol, (**b**) Nyquist plot of EIS and the simulation using an equivalent circuit model for the electrolytes containing 0, 5, and 10% v/v ethanol.

Fig. 6a shows the Nyquist plots of the electrolytes containing 0–50% v/v ethanol. The Nyquist plots exhibit a semicircle in the high frequency (charge transfer resistance, *R*_*ct*_). The charge transfer resistance of 5 and 10% v/v ethanol electrolytes are much smaller than that of 0% v/v ethanol electrolyte, confirming that the charge transfer resistance is significantly reduced by adding ethanol to KOH solution. However, the charge transfer resistance increased at higher concentration of ethanol electrolyte above 40% v/v. The smaller *R*_*ct*_ indicates that the surface reaction is faster than that of 0% v/v ethanol electrolyte, which is more beneficial to the charge/discharge process. Consistently with the CV results, the charge transfer resistance decreased when ethanol was added to KOH solution less than 20% v/v, confirming an increase of zincate formation due to ethanol introduced. The charge transfer resistance of 5% v/v ethanol is the smallest, which implies a highly efficient electron transport for oxidation reaction.

Also, the Nyquist plots of the electrolytes containing 0, 5, and 10% v/v ethanol were modelled with an electrical equivalent circuit model as shown in Fig. 6b. The model includes resistant *R*_*s*_, constant phase element CPE, charge transfer resistant *R*_*ct*_ and Warburg impedance *W*. In all cases, the shapes of the Nyquist plots were similar indicating that there are no differences in zinc dissolution mechanism, and the process is charge transfer controlled. The model includes the double layer capacitance *C*_*dl*_ is placed in parallel to the charge transfer resistance *R*_*ct*_ with a small inclined line at low frequencies related to the Warburg impedance associated with the diffusion of soluble ions in the bulk electrolyte. The double layer capacitance is replaced by a constant phase element (CPE) representing a frequency distributed double layer capacitance with a phase shift (*n*). The impedance of the CPE is given in (10).

10 $${C}_{dl}=Q{\mathrm{(2}\pi {f}_{{\rm{\max }}})}^{n-1}$$

The parameters of the equivalent circuit used are shown in Table 2. *R*_*s*_ represents the solution resistance that increases with addition of ethanol because of the lower polarity of ethanol compared to water. The diameter of semi-circle increases with the order of 0% v/v ethanol ≥ 10% v/v ethanol ≥ 5% v/v ethanol. The smaller *R*_*ct*_ are observed for 5% v/v ethanol as compare to the others. The 0% v/v ethanol electrolyte revealed a decrease of the double layer capacitance (*C*_*dl*_) and an increase in the charge transfer resistance (*R*_*ct*_) at the electrode/electrolyte interface. The double-layer capacitance depends on different parameters such as ionic concentrations, electrode potential, types of ions, temperature, electrode roughness, oxide layers, impurity adsorption, etc. *C*_*dl*_ of the electrolytes containing ethanol is lower than that of 0% v/v ethanol electrolyte. The increase of *C*_*dl*_ may be resulted from specific adsorption of ethanol and ethoxide ions on the zinc surface.

**Table 2:** EIS parameters obtained by fitting the data to equivalent circuit models.

	*R*_s_	*Q*,CPE	*n*	*C*_*dl*_	*R*	*W*	*χ* ^2^
	Ω	S.s^*n*^	0 < n < 1	F	Ω	S.s^5^	error
0% v/v	0.309	1.12E-3	0.701	3.03E-4	3.067	0.927	2.74E-3
Ethanol							
5% v/v	0.389	9.80E-2	0.725	1.56E-2	0.780	6.392	1.46E-4
Ethanol							
10% v/v	0.462	6.60E-3	0.800	2.29E-3	1.822	0.038	2.17E-4
Ethanol							

**Figure 7:**
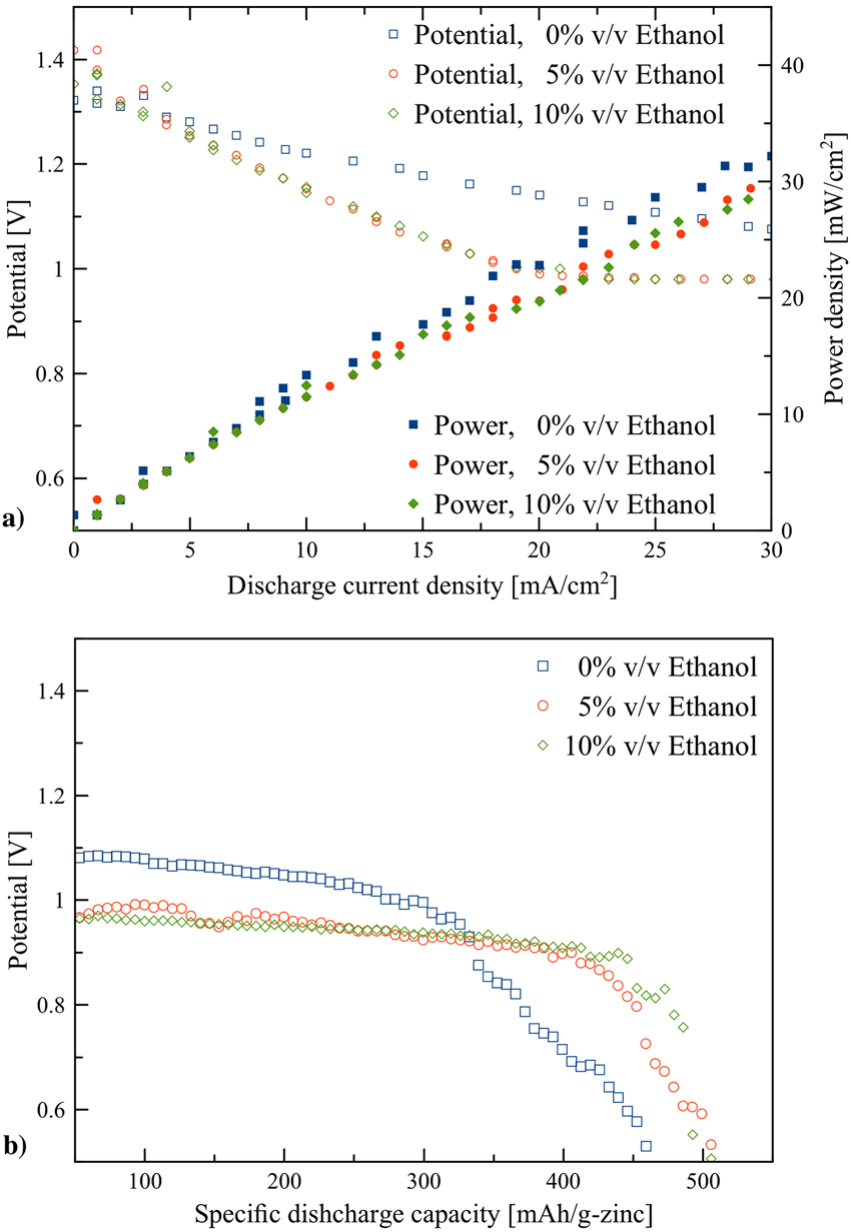
Performances of the zinc-air flow batteries with the electrolyte circulation rate of 20 mL/s: (**a**) polarization characteristics, and (**b**) galvanostatic discharge profiles of the batteries using 0, 5, and 10% v/v ethanol.

The flow batteries were fabricated using the anode made of zinc granules. The flow rate of the electrolytes was set at a circulation rate of 20 mL/min. The polarization characteristics of the batteries with the electrolytes containing ethanol 0%, 5%, and 10% v/v are shown in Fig. 7a. Also, the relationship between discharge power and discharge current of the batteries is also shown Fig. 7a. The polarization characteristic for each case was similar indicating that the same chemical reactions and phenomena occurred. In general, a high discharge potential at high discharge current is desirable. Nevertheless, all cases revealed a linear potential drop with an increase of discharge current, indicating that ohmic losses dominated the cell performance. The addition of ethanol showed adverse effects on polarization characteristics of the batteries by demonstrating relatively greater potential drop.

The performance of the batteries is further examined at a constant discharge current density of 25 mA/cm^2^. Figure 7b presents the galvanostatic discharge profiles of the batteries. In all cases, the discharge profiles were similar to typical discharge profiles for zinc-air batteries using porous zinc anode^40^ or a compact zinc plate anode^41^. Ethanol has a substantial effect on the discharge profile. Though, 5% and 10% v/v ethanol electrolytes exhibited relatively lower discharge potential, the discharge period is much longer, confirming the positive effect of ethanol on the discharge performance. The battery with 0% v/v ethanol electrolyte yielded the lowest specific capacity of 360 mAh/g (494 mWh/g) at a cut-off voltage of 0.8 V. By adding 5% v/v ethanol, the batteries exhibited 450 mAh/g and 548 mWh/g (25% improvement in specific capacity and 11% improvement in specific energy). In comparison, the battery using 10% v/v ethanol electrolyte exhibited 470 mAh/g and 576 mWh/g (30% improvement in specific capacity and 16% improvement in specific energy). The batteries with the electrolytes containing ethanol showed significantly higher discharge capacity and energy in comparison with the battery without ethanol.

## Conclusion

This work demonstrated the positive effects of the addition of ethanol to 8 M KOH aqueous solution as the electrolyte in zinc-air flow batteries. The utilization of ethanol was studied for a range of different concentrations ethanol (0–50% v/v). Cyclic voltammograms, electrochemical impedance spectroscopy and potentiodynamic polarization measurements showed that the presence of 5–10% v/v ethanol is attributed to the enhancement of zinc dissolution and the hindrance of passivation of the zinc anode. Thus, 5–10% v/v ethanol electrolytes provided the highest electrochemical performance. Besides, the galvanostatic discharge results showed that the battery with 10% v/v ethanol electrolyte exhibited the highest improvement over that of 0% v/v ethanol electrolyte with 30% increase in discharge capacity and 16% increase in specific energy. The proposed approach is simple yet effective approach to improve performances of zinc-air batteries and can also be implemented in other zinc-based alkaline batteries.

### Data Availability

The authors declare that all relevant data are within the paper.
